# A patient with ROHHAD syndrome and leptin-melanocortin pathway gene variations. Correlation or just a coincidence? Case report

**DOI:** 10.1007/s42000-025-00754-z

**Published:** 2026-01-28

**Authors:** Eirini Kostopoulou, Alexandra Efthymiadou, Aristeidis Giannakopoulos, Dionisios Chrysis

**Affiliations:** 1 Division of Pediatric Endocrinology and Diabetes, Department of Pediatrics, University General Hospital of Patras, Patras, 26500 Greece; 2 University of Patras, 26504 Patras, Greece

**Keywords:** ROHHAD syndrome, Syndromic obesity, Monogenic obesity, PLXNA3, PCSK1

## Abstract

**Purpose:**

In cases of early onset, severe obesity, especially when in the presence of additional clinical manifestations, genetic investigation to exclude syndromic or monogenic obesity is indicated. ROHHAD syndrome is one of the rarest syndromes associated with obesity. Genetic analysis has not to date identified responsible genes; therefore, its diagnosis is based on clinical criteria. Monogenic obesity is mainly caused by variants in genes involved in the leptin-melanocortin pathway, the satiety pathway.

**Case presentation:**

We present a patient with severe obesity from the age of 3 years, endocrinopathies (central diabetes insipidus, central hypothyroidism, precocious puberty, and growth hormone deficiency), hypoventilation, cold extremities, psychomotor retardation, behavioral disorders, strabismus, fatty liver infiltration, and syndromic facial features. Based on the clinical features, ROHHAD syndrome was diagnosed. The patient underwent genetic testing of 80 genes involved in the leptin-melanocortin pathway. He was found to be heterozygous in the *PLXNA3* gene for a sequence variant designated c.3640 C > T, which is predicted to result in the amino acid substitution p.Arg1214Trp. This variant has not been reported in the literature and is of unknown clinical significance. The patient was also heterozygous in the *PCSK1* gene for a sequence variant designated c.661 A > G, which is predicted to result in the amino acid substitution p.Asn221Asp and is classified as a risk factor for obesity.

**Conclusion:**

We present the first reported patient with ROHHAD syndrome and variants in two genes involved in the leptin-melanocortin pathway. The possibility of the coexistence of two rare types of obesity, syndromic and monogenic, makes this case particularly exceptional and raises questions about the potential association between the two entities.

## Introduction

The most common form of childhood obesity is multigenic obesity, which is due to the cumulative effect of many genes. However, in cases of early onset, severe obesity, especially when it is characterized by accompanying clinical manifestations, such as endocrinopathies, syndromic features, psychomotor retardation, neurological disorders, or behavioral problems, genetic investigation to exclude syndromic or monogenic obesity is indicated [1]. Syndromic obesity is usually caused by gene variants or chromosomal translocations resulting in early onset, severe obesity, dysmorphic features, intellectual or developmental delay, and multisystem manifestations. The two commonest syndromes that are associated with severe obesity are Prader Will syndrome and Bardet Biedl syndrome [1]. Another very rare form of syndromic obesity is ROHHAD syndrome, a relatively new multisystemic disorder of unknown etiology with high morbidity and mortality (50–60%) [2]. Approximately 200 cases have been reported to date worldwide. It is characterized by a sequence of clinical features, including rapid onset obesity, hypothalamic dysfunction, hypoventilation, and autonomic dysregulation, which usually, but not necessarily, emerge in the order of the acronym [2, 3]. The disease is diagnosed when all the above clinical criteria are present in the absence of alternative possible diagnoses [2].

Monogenic obesity is also characterized by early onset, severe obesity, and insatiable hunger, also known as hyperphagia. It is caused by rare pathogenic variants in single genes involved in the leptin-melanocortin pathway (satiety pathway), which exert strong effects, and is highly penetrant. The contribution of gene variants involved in the leptin-melanocortin pathway to the obesity phenotype is a rapidly evolving field.

We report the case of a patient with co-occurrence of ROHHAD syndrome, one of the rarest causes of syndromic obesity, and two variants in genes associated with monogenic obesity, another particularly rare type of obesity. In parallel, we conducted a literature review summarizing the clinical features of ROHHAD syndrome and the available treatment options.

The research complies with the guidelines for human studies and was conducted ethically in accordance with the World Medical Association Declaration of Helsinki. Written informed parental consent was obtained from the patient’s parents for publication of this case report and any accompanying images.

## Case presentation

A 6-year-old female was referred to the Pediatric Endocrine Clinic of a tertiary university hospital due to early onset, severe obesity and severe hyperphagia from the age of 2 years. She is the second child of unrelated healthy parents, born at full term, with a birth weight of 3,000 g. There was no relevant family history. On the first day of life she developed hypoglycemia that resolved within the following 24 h. At the age of 3 months, she was hospitalized due to hypernatremia that emerged over the course of a febrile illness.

The patient also had psychomotor and developmental delay. She sat at the age of 8 months, walked at the age of 4 years, and spoke at the age of 5 years. At the age of 6 years, she had not yet acquired sphincter control. She also had mild intellectual disability, learning difficulties, and behavioural disturbances. Polyuria and polydispia were also present. Family history was unremarkable and the parents and brother had normal BMI values.

**Fig. 1:**
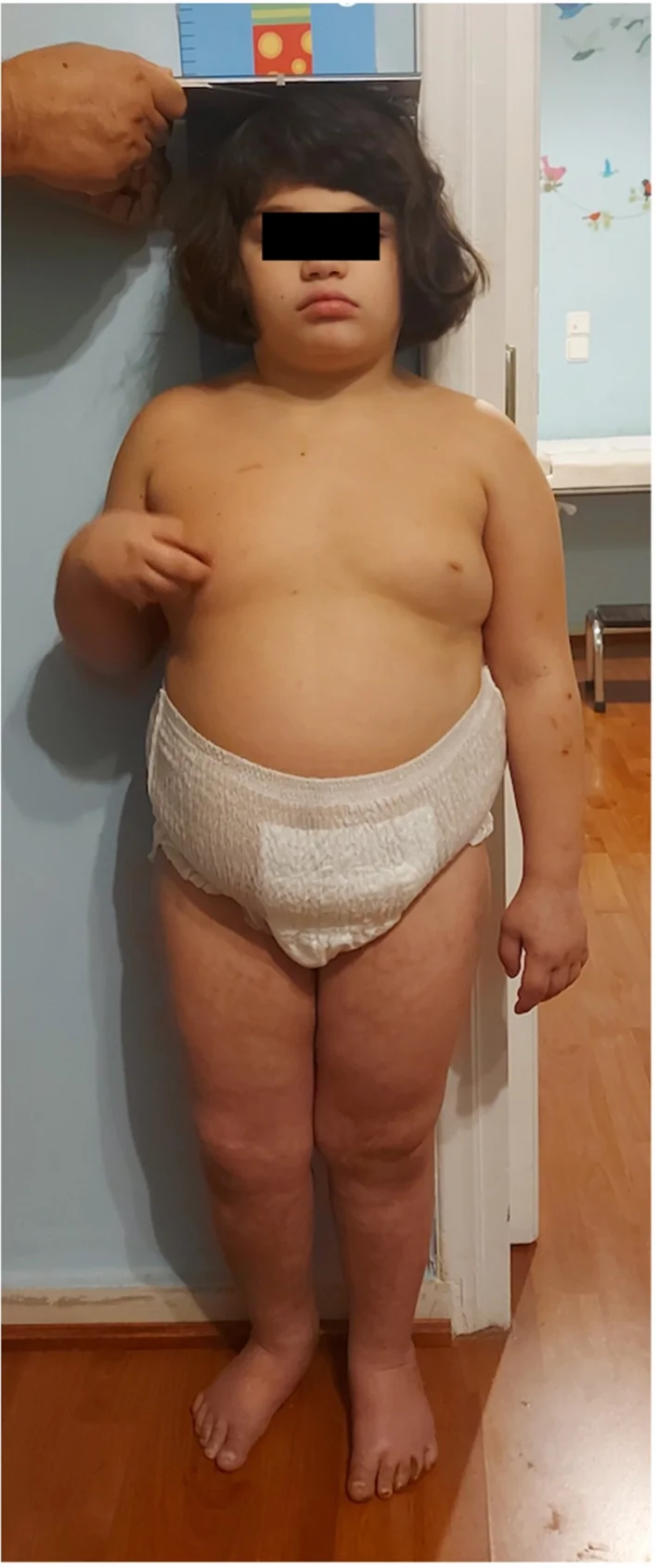
Patient with ROHHAD syndrome and genetic variations in the leptin-melanocortin pathway

**Fig. 2:**
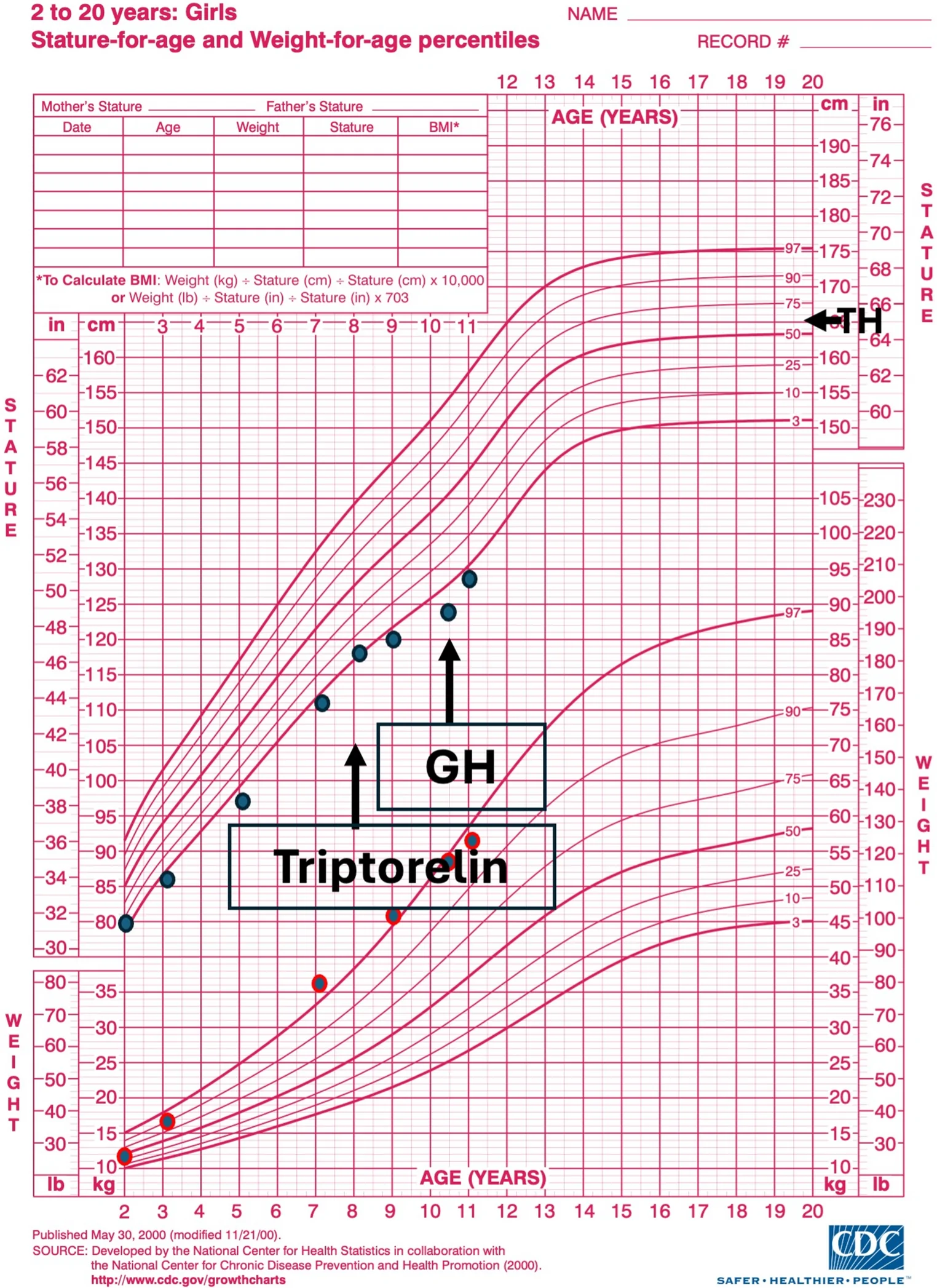
Patient’s growth charts (weight and height). The time of initiation of triptorelin and growth hormone treatment are depicted

**Fig. 3:**
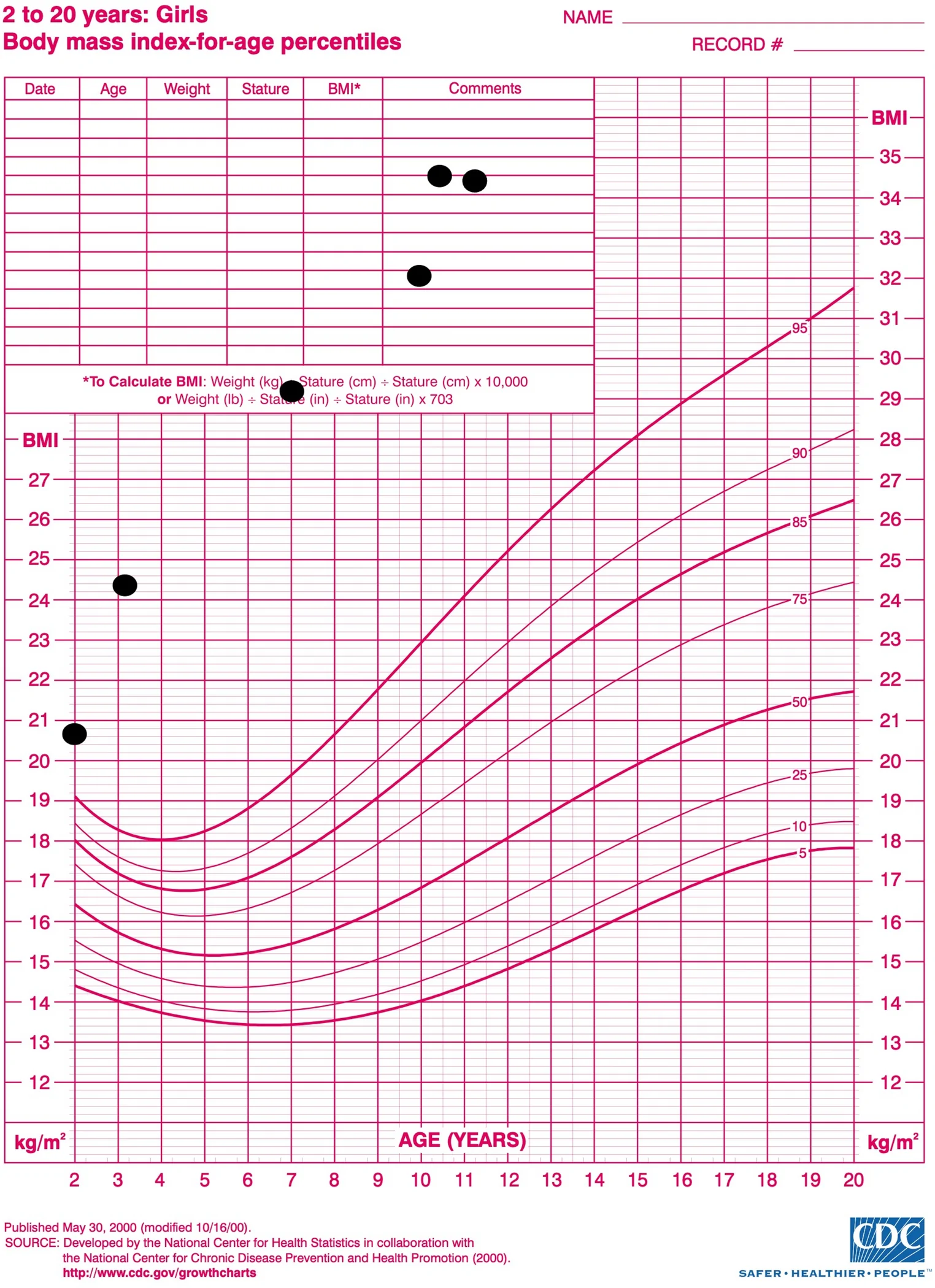
Patient’s BMI chart

On physical examination, syndromic facial features were recognized, such as depressed nasal bridge, broad nasal tip and down-turned mouth (Fig. 1)**.** Strabismus (eye misalignment), hepatomegaly, red and cold extremities, and livedo reticularis in the upper and lower extremities were also observed. Her pubertal stage was Tanner II, while the growth charts revealed short stature (< 3rd percentile for age and sex) and rapid weight gain after the age of 2 years (Figs. 2 and 3).

Biochemical blood investigations showed hypertransaminasemia and hypernatremia (Na: 147 mg/dl) (Table 1). Due to polyuria and polydipsia, persistent hypernatremia, low urine specific gravity (1003), and a urine output of > 4 ml/kg/h, a desmopressin test was performed which confirmed the diagnosis of central diabetes insipidus. Hormonal investigations showed pubertal levels of LH and FSH (Table 1) and activation of the hypothalamic-pituitary-gonadal axis (max LH: 11.8) was confirmed on an LHRH test (Table 1)**.** In parallel to precocious puberty, central hypothyroidism was diagnosed at the age of 7.5 years due to inappropriately normal TSH concentrations for the low FT4 levels measured on two occasions (Table 1)**.**

**Table 1:** Blood investigations performed in the patient

Biochemical investigations (6 years old)			
WCC (/mm^3^): 8.900		Κ (mEq/L): 4.1	
Hb (g/dl)/Hct (%): 12.1/36.7		**Νa (mEq/L): 147**	
PLT (/mm^3^): 205.000		HbA1c (%): 5.2	
Glucose (mg/dl): 77		Total cholesterol (mg/dl): 181	
Urea (mg/dl): 22		LDL (mg/dl): 110	
Creatinine (mg/dl): 0.51		HDL (mg/dl): 43	
**AST (U/l): 49**		Triglycerides (mg/dl): 142	
**ALT (U/l): 139**			
**Endocrine investigations (6 years old)**			
**TSH (µIU/ml): 2.05 − 4.36**		Insulin (µIU/ml): 12.9	
**FT4 (ng/ml): 0.88 − 0.665**		HbA1c (%): 5.2	
Anti-TG: 13.45		**LH (mIU/ml): 0.8**	
Anti-TPO: 11.2		**FSH (mIU/ml): 4.82**	
Cortisol (µg/dl): 11.2		Estradiol (pg/ml): <5	
ACTH (pg/ml): 43.36		Prolactin (ng/ml): 9.62	
PTH (pg/ml): 41.79		25(OH)VitD (ng/ml): 20.6	
**IGF-1 (ng/ml): 61.9 (5**^**η**^ **ΕΘ)**		β-hCG µIU/ml: 0.1	
**LHRH test (6 years old)**			
**T (min)**	**0**	**30**	**60**
**LH (mIU/ml)**	0.2	**4.8**	**5.4**
**FSH (mIU/ml)**	3.7	9.5	12
**LHRH test (7.5 years old)**			
**T (min)**	**0**	**30**	**60**
**LH (mIU/ml)**	**1.2**	**11.4**	**11.8**
**FSH (mIU/ml)**	5.5	12.3	14.1
**Thyroid function tests (7.5 years old)**			
**TSH (** **μ** **IU/ml)**	**4.36**		
**FT4 (ng/ml)**	**0.665**		

*WCC* white cell count, *Hb* hemoglobin, *Hct* hematocrit, *PLT* platelets, *AST* aspartate aminotransferase, *ALT* alanine aminotransferase, *HbA1c* hemoglobin A1c, *LDL* low-density lipoprotein, *HDL* high-density lipoprotein, *TSH* thyroid-stimulating hormone, *FT4* free thyroxine, *anti-TG* anti-thyroglobulin antibodies. *anti-TPO* anti-thyroid peroxidase antibodies, *ACTH* adrenocorticotropic hormone, *PTH* parathyroid hormone, *IGF-1* insulin growth factor 1, *LH* luteinizing hormone, *FSH* follicle-stimulating hormone, *β-hCG* β-human chorionic gonadotropin

An oral glucose tolerance test was normal, whereas an abdominal ultrasound revealed metabolic dysfunction-associated steatohepatitis (MASH).

Further examinations included a normal karyotype, negative tests for Prader Willi syndrome, and Cushing’s syndrome. ENT and pulmonary assessments were unremarkable and ophthalmological assessment revealed abducens nerve palsy, possibly in the context of Duane retraction syndrome. An MRI head scan showed a small pituitary gland and absent posterior pituitary bright spot. A large cystic formation was also identified at the level of the quadrigeminal cistern, with dimensions of 31 × 24 × 21 mm. The differential diagnosis primarily includes the presence of an arachnoid or a giant epiphyseal cyst.

At the age of 7.5 years, her growth velocity declined, IGF-1 was low (22 ng/ml, < 0.1st percentile for age), and the GH provocation tests (clonidine and L-DOPA tests) confirmed GH deficiency (max GH: 1.49 ng/ml and 2.80 ng/ml, respectively). Due to significant central and obstructive sleep apneas during a polysomnography (PSG), growth hormone replacement therapy was not initiated. BiPAP treatment was started and, at the age of 10.5 years, a repeat PSG showed no apneas on BiPAP, allowing for growth hormone treatment to be started. Bone age at initiation of growth hormone treatment was 12 years. In total, the patient received treatment with desmopressin, triptorelin, L-thyroxine, somatropin, and BiPAP. Her weight status deteriorated persistently, with the BMI z-scores increasing from 1.47 at the age of 2 to 3.1 at the age of 3, 5.14 at the age of 7, 5.7 at the age of 9, and 6.1 at the age of 11 years.

Based on clinical criteria, the patient was diagnosed with ROHHAD syndrome. However, due to the coexistence of early onset, severe obesity and neurodevelopmental disorders, but also due to the unknown association between the syndrome and genetic causes, genetic testing for monogenic obesity was performed using a combination of Next Generation Sequencing and Sanger sequencing. The patient was found heterozygous in the *PLXNA3* gene for a sequence variant designated c.3640 C > T, which is predicted to result in the amino acid substitution p.Arg1214Trp. To our knowledge, this variant has not been reported in the literature and is of unknown clinical significance.

The patient was also heterozygous in the *PCSK1* gene for a sequence variant designated c.661 A > G, which is predicted to result in the amino acid substitution p.Asn221Asp: it is therefore classified as a risk factor for obesity.

## Discussion

We present a female with early onset, severe obesity that fulfils the clinical criteria for ROHHAD syndrome, i.e., rapid onset obesity, hypothalamic dysfunction, hypoventilation, and autonomic dysregulation (Table 2). In patients with the syndrome, rapidly progressive obesity is usually the first recognized clinical sign, with the onset ranging from 0 to 9 years and most frequently being between 2 and 4 years. Hyperphagia is also usually present. Hypothalamic dysfunction manifests as multiple hormonal deficiencies, such as central hypothyroidism and growth hormone deficiency, whereas the principal hormonal dysregulation reported is hyperprolactinemia [4]. Dysnatremia, either hyponatremia or hypernatremia due to SIADH or diabetes insipidus, respectively, are also frequently observed. Central hypoventilation is caused by abnormal response to hypoxia and hypercapnia due to a dysfunction of the autonomic nervous system and can be potentially fatal due to cardiorespiratory arrest. Autonomic dysregulation, also known as dysautonomia, includes gastrointestinal, ophthalmic and cardiological symptoms, thermic dysregulation, altered perception of pain, dysarthria, and pulmonary hypertension (Table 2).

**Table 2:** Clinical features of ROHHAD syndrome

Onset of the disease: 0–9 years (usually 2–4 years)	Neurologic disorders (seizures, sleep disturbance, hypersomnolence, narcolepsy, developmental delay, gait disturbance, nystagmus, general weakness
**Rapid and early onset**,** severe obesity**,** hyperphagia**	**Dysmorphic features** (**depressed nasal bridge**, macrocephaly, anteverted nares, hypertelorism)
**Hypothalamic dysfunction** (**growth hormone deficiency**,** diabetes insipidus**, transient SIADH, hypodipsia, **central precocious puberty**, precocious adrenarche, amenorrhea, hypogonadotropic hypogonadism, hyperprolactinemia, **hypothyroidism**, ACTH deficiency, **dysnatremia**	Tumors of neural crest origin (40–56% of patients, gaglioneuroma or gaglioneuroblastoma in the chest, abdomen, or along the sympathetic nervous system chain).
**Autonomic dysregulation**: blurred vision, **strabismus**, ptosis, altered pupil response to light, altered perception of pain, gastrointestinal dysmotility with constipation or diarrhea, bradycardia, neurogenic bladder, excessive sweating, thermal dysregulation, **cold hands and feet**, pseudo-Raynaud’s phenomenon, syncope, urinary incontinence, dysarthria, pulmonary hypertension	**Metabolic disorders** (insulin resistance, impaired glucose tolerance, diabetes mellitus, hypertriglyceridemia, fatty liver **disease**)
**Hypoventilation** (**obstructive sleep apnea**, hypoxemia, hypercapnia, **central sleep apnea**, abnormal ventilatory response to carbon dioxide, nocturnal hypoventilation, cyanotic episodes	**Behavioral disorders** (**mood changes**, irritability, aggression, fatigue, **social withdrawal**,** poor school performance**,** intellectual disabilities**, hallucination, major depressive disorder, **self-injurious behavior**,** obsessive-compulsive disorder**, bipolar disorder, psychosis

The patient’s clinical features are shown in bold.

Psychiatric symptoms (e.g., hallucinations, psychosis, and major anxiety) and behavioral disorders (e.g., aggressiveness, hyperactivity, and irritability) have also been described in patients with the syndrome [4]. In more than half of the cases, neural crest tumors (NETs) are present (ROHHADNET), manifesting usually within the first 2 years from the onset of obesity. The most frequent types of NETs are ganglioneuroblastoma, neuroblastoma, and ganglioneuroma [4].

Genetic, epigenetic, and immune-modulated etiopathogenic theories have been postulated. No association has however been found between the syndrome and any of the investigated genes to date [2]. Therefore, the diagnosis remains a challenge as the etiology is obscure, there are no confirmatory tests, and the diagnosis relies on clinical criteria alone. Treatment is supportive and addresses the clinical presentations. It includes management of obesity, hormone replacement, artificial ventilation through mask ventilation, continuous positive airway pressure, mechanical ventilation or tracheostomy, antihypertensives, stool softeners, antidiarrhea medications, antiepileptics, antipsychotics, and antidiabetic and hypolipidemic medications. In the presence of neural crest tumors, surgical removal is performed and multimodal treatment is conducted [2].

The patient was also found positive for variants in two genes, *PLXNA3* and *PCSK1*, that are involved in the leptin-melanocortin pathway and have been associated with monogenic obesity.

The *PLXNA3* gene encodes a plexin member protein (plexin A3), which is a semaphorin type 3 receptor. Semaphorins are axon guidance molecules that participate in the development of the nervous system and apoptosis and that regulate the morphology and motility of different cell types. Variants in the *PLXNA-3* gene are thought to be involved in the migration of POMC neurons from the arcuate to the paraventricular nucleus and have been reported in patients with obesity, autism spectrum disorder, schizophrenia, and hypogonadotropic hypogonadism [5]. Variants in *PLXNA3* have also been reported in patients with intellectual disability [6], autism spectrum disorder, and obesity [5]. However, since few cases have been described, these gene-disease associations are considered provisional at this time. Rare variants in the *PLXNA3* gene have been found enriched in individuals with severe, early-onset non-syndromic obesity, contributing to the obesity through an oligogenic mechanism. In patients with hypogonadotropic hypogonadism, obesity is a common accompanying feature; however, it is considered a comorbidity rather than a primary syndromic feature [7]. The presented case is the first to report a variant in the *PLXNA3* gene in the context of syndromic obesity, which underscores the novelty of the case. The variant in the *PLXNA3* gene that was detected in our patient is reported in 0.0080% of alleles in individuals of African descent in gnomAD and has been observed in the hemizygous state in two male individuals [8]. At the present time, the clinical significance of this variant is uncertain due to the absence of conclusive functional and genetic evidence. Nonetheless, the neurocognitive abnormalities observed in our patient might be related to the variant since developmental delay is not a hallmark feature of ROHHAD syndrome.

The protein encoded by the *PCSK1* gene, proprotein convertase 1/PC1, catalyzes the cleavage of prohormones into their functional forms, including proopiomelanocortin/POMC to ACTH and α-melanocyte-stimulating hormone/MSH. This cleavage is an important step in the activation of the leptin-melanocortin pathway. Variants of the *PCSK1* gene have been associated with morbid obesity, endocrine disorders, hypoglycemia, and malabsorptive diarrhea [9]. The variant found in the *PCSK1* gene is common in the general population and classified as benign for Mendelian inheritance of obesity. However, multiple studies have demonstrated that the p.Asn221Asp substitution causes a modest decrease in secreted enzyme levels and catalytic activity [10–12]. In addition, a meta-analysis of multiple association studies concluded that heterozygosity for the c.661 A > G. polymorphism (rs6232) is associated with body mass index as a quantitative trait and with binary obesity status [13]. Taken together, the variant is classified as a risk factor for obesity. We have previously described a patient with Prader-Willi syndrome and the same variant in the PCSK1 gene [14]. Although this is a common genetic variant and a polygenic risk factor for obesity, it might also be implicated in syndromic obesity through an unknown pathophysiological mechanism or solely by enhancing the severity of obesity. This is the second reported case of co-occurrence of syndromic obesity and this variant.

In conclusion, in cases of early-onset, severe obesity and concomitant features (e.g., endocrine disorders or developmental delay or behavioral disorders), the etiology of obesity may be complex and multifactorial. In such cases, the presence of an obvious diagnosis of obesity does not exclude the need for a second diagnosis, which can be either related to the first or not. The development and spread of genetic methods, and thus better access to genetic investigation, offer new knowledge that often overturns our previous understanding of the causes of severe obesity and reveals that monogenic obesity is not as rare as previously thought. Furthermore, the existence of genetic variants in genes of the leptin-melanocortin pathway should be suspected in cases of early-onset, severe obesity accompanied by concomitant clinical manifestations, even in the presence of a known diagnosis. The combination of more than one genetic variant, each of which is not sufficient to explain the phenotype, may have a cumulative pathogenic effect through synergy. Of particular importance, the identification of specific genetic defects affecting the satiety pathway or neuronal migration may explain the phenotypical variability seen in patients with syndromic obesity. It may also offer new, targeted therapeutic options for monogenic obesity, such as setmelanotide, an MC4R agonist, which, is the first and currently only approved treatment targeting the MC4R pathway.

This is the first reported case of a patient with ROHHAD syndrome with coexisting variants in genes involved in the leptin-melanocortin pathway. Although the relationship of these variants to the patient’s full phenotype cannot be demonstrated based on the current knowledge, their presence cannot be overlooked as simply coincidental. The possibility of the co-occurrence of syndromic and monogenic obesity, two particularly rare forms of obesity, renders this case particularly noteworthy. The possibility of an association between the two entities requires further investigation.

## Data Availability

The data that support the findings of this study are available from the corresponding author upon reasonable request.
